# Convulsive status epilepticus in an emergency department in Cameroon

**DOI:** 10.1016/j.ebr.2021.100440

**Published:** 2021-03-22

**Authors:** Daniel Gams Massi, Christophe Davy Endougou Owona, Annick Mélanie Magnerou, Albert Justin Kana, Seraphine Mojoko Eko, Jacques Doumbe, Njankouo Yacouba Mapoure

**Affiliations:** a Douala General Hospital, PO Box: 4856, Douala, Cameroon; b Faculty of Health Sciences, University of Buea, PO Box: 63, Buea, Cameroon; c Faculty of Medicine and Pharmaceutical Sciences, University of Douala, PO Box: 2701, Douala, Cameroon

## Abstract

• In Cameroon, the most common cause of CSE was stroke followed by infection.
• Despite resource limitations, developing countries can effect protocols for CSE.
• Despite limited antiseizure medications, outcomes were similar in Cameroon to multicenter outcomes.

## Introduction

In Africa, few epidemiological data are available on status epilepticus (SE). Studies carried out in sub-Saharan Africa have found a hospital-based prevalence varying between 5.28 and 10.86% [1], [2]. Clinically there are two presentations: the convulsive SE (CSE) and the non-convulsive SE (NCSE) [3]. The commonest causes are: cerebrovascular accidents, antiseizure medications (ASM) disruptions, and central nervous system (CNS) infections [4]. In Africa, the most frequent etiologies are vascular and infectious [2], [5], [6], [7]. Current management recommendations are based on studies from developed countries [8]. In Cameroon, a resource-limited African country, the scarcity of ASM pointed out in the international recommendations and the restrictions in diagnostic tools, leaves very few therapeutic possibilities for the best management of patients suffering from SE. This study aimed to determine the prevalence of CSE, to describe the clinical and etiological pattern, and evaluate the outcome of CSE in a tertiary health care center in Cameroon.

## Material and methods

### Study design

The Douala General Hospital is the main referral health facility in the city of Douala (economic capital of Cameroon). This hospital comprises a Medico-Surgical Emergency Department (MSED) which functions 24/7. This cross-sectional study was carried out from December 2019 to May 2020 in the MSED. Ethical approval was obtained from the Institutional ethics committee of the University of Douala (N ° 2141/CEI-UDo/01/2020/T).

### Participants

Patients included in this study were of age 16 and above, admitted to the MSED for CSE. The 16-year cut-off corresponds to the age below which patients are rather admitted to pediatric emergency unit. Operationally, CSE has been defined as a seizure lasting more than five minutes or the succession of at least two seizures without recovery of consciousness in-between. Refractory SE (RSE) was defined as the persistence of seizure after administration of first and second line of ASM.

### Data collection

The data were collected on a pre-test questionnaire which contained socio-demographic data, past medical history, clinical data such as CSE description, duration of seizures, etiologies, treatment received, and outcome. The informed consent was obtained from the patient or the family (when patient was unconscious) before any data collection.

### Statistical analysis

Data were entered and analyzed with SPSS 20.0 software (SPSS, IBM SPSS Statistics, New York, USA). Continuous variables were expressed as mean ± standard deviation (SD). Categorical variables were presented as frequency (percentage) or by graphical representation. Univariate analysis was performed to determine factors associated to mortality. The results were reported as adjusted Odd ratio (OR) with 95% confidence interval (CI). The significance level was set at p < 0.05.

## Results

During the study period, 2,601 patients were received at the MSED of the DGH, including 53 cases of CSE (hospital-based prevalence of 2.03%). The mean age was 49.19 ± 18.07 years, with males representing 62.3% of cases. Patients were mainly located in Douala (94.3%) and only 17% of cases came to the hospital in an ambulance. Patients with epilepsy accounted for 26.4% of cases, and 57.1% of them were on ASM. Focal CSE was found in 54.7% of patients. Seizures lasted at least 30 min in 22.6% of cases. Brain imaging (CT scan or MRI) was performed in 92.4% of patients. Etiologies were led by stroke followed by CNS infections (Table 1).

**Table 1:** Etiologies of convulsive status epilepticus.

Etiologies		n	%
Stroke	Ischemic stroke	7	13.2
	Intracerebral hemorrhage	4	7.5
	Sub-arachnoid hemorrhage	2	3.8
CNS infections	Cerebral toxoplasmosis	4	7.5
	Bacterial meningitis	2	3.8
	Cryptococcal meningitis	1	1.9
	Viral encephalitis	1	1.9
	Severe malaria	1	1.9
ASM modifications	ASM withdrawal	7	13.2
	ASM change	1	1.9
Brain tumor	Meningioma	2	3.8
	Cavernoma	2	3.8
	Oligodendrioglioma	1	1.9
	Cerebral metastasis	1	1.9
	Glioblastoma	1	1.9
Brain trauma	Acute sub-dural hematoma	4	7.5
	Hemorrhagic contusion	2	3.8
	Chronic sub-dural hematoma	1	1.9
Metabolic disorders	Hypoglycemia	3	5.7
	Uremic syndrome	3	5.7
	Hyperglycemia	1	1.9
	Eclampsia	1	1.9
Unknown		1	1.9

ASM: antiseizure medication; CNS: central nervous system.

**Fig. 1:**
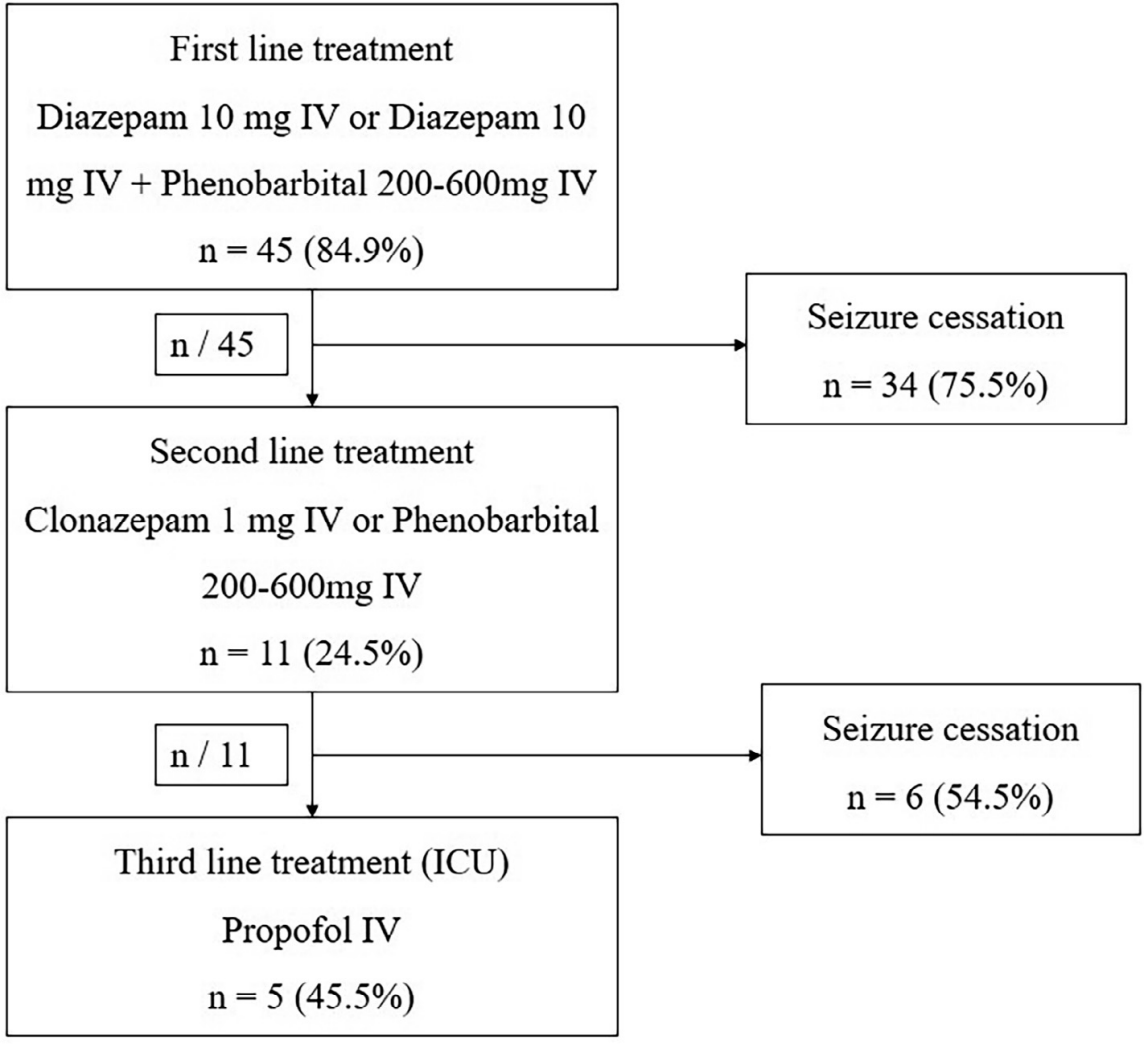
Treatment protocol administered to patients with CSE at their admission in the MSED of the Douala General Hospital.

Concerning the treatment, 84.9% (n = 45) of patients received first-line treatment containing (either Diazepam 10 mg IV repeated once if necessary) with seizure cessation in 75.5% (n = 34) of these cases. Eight patients reached the MSED just after seizure cessation and were not exposed to any ASM. In the second line treatment, 20.7% (n = 11) of patients received either Clonazepam 1 mg IV (n = 7) or Phenobarbital 200–600 mg (n = 3). After the second line treatment, 9.4% (n = 5) of patients developed RSE and were admitted in the intensive care unit (ICU) on Propofol (Fig. 1).

The mean duration of hospitalization (MDH) was 11.7 ± 5.8 days. And the mean time from admission to death was 4.1 ± 2.9 days (Table 2).

**Table 2:** Time and duration important for management of patients.

Period	Mean (SD)
Time from admission to GP consultation (minutes)	10,1 (3.8)
Time from admission to first treatment (minutes)	8,2 (3.6)
Time from admission to first line ASM (minutes)	12,1 (6.2)
Time from admission to brain imaging (hours)	3,5 (1.6)
Duration of hospitalization (days)	11.7 (5.8)
Time from admission to death (days)	4.1 (2.9)

GP: general physician.

In-hospital mortality was 16.9% (n = 9), and RSE was significantly associated to death (*p* = 0.03; OR: 10.5 (CI 1.44–76.28). Details on univariate analysis are found on Table 3.

**Table 3:** Univariate analysis to determine factors associated to mortality.

	Variables	n (%)	p-value	OR (IC 95%)
Age groups	≤60	33 (56.6)	–	Ref
	>60	20 (37.7)	0.05	4.61(0.99–21.33)
Gender	Male	33 (62.2)	0.71	1.40(0.32–5.97)
Preexisting epilepsy	Yes	14 (26.4)	0.99	0.76(0.13–4.19)
CSE type	Generalized CSE	24 (45.3)	0.16	0.28(0.53–1.53)
	Focal CSE	29 (54.7)	0.16	3.50(0.65–18.75)
Seizure duration	<30 min	41 (77.4)	0.4	1.94(0.40–9.32)
	≥30 min	12 (22.6)	-	Ref
	**RSE**	**5 (9.4)**	**0.03**	**10.50(1.44**–**76.28)**
Etiologies	Ischemic stroke	7 (13.2)	0.58	2.22(0.35–13.84)
	Hemorrhagic stroke	4 (7.5)	0.99	0.97(0.10–9.51)
	CNS infections	9 (16.9)	0.33	-
	ASM modifications	9 (16.9)	0.32	-
	Brain tumor	7 (15.1)	0.58	2.22(0.35–13.84)
	Metabolic disorders	8 (13.2)	0.58	2.22(0.35–13.84)
	Brain trauma	7 (13.2)	0.99	0.79(0.08–7.51)
	Unknown cause	1 (1.8)	0.17	-

ASM: antiseizure medication; CSE: convulsive status epilepticus; RSE: refractory status epilepticus.

## Discussion

This hospital-based study on CSE represents the first interest on this topic in our setting. The prevalence of 2.03% found is lower than the prevalence reported in other Sub-Saharan African (SSA) countries [1], [2], [9]. Our study was carried out in an emergency department receiving patients from different specialties, whereas other SSA studies had been carried out exclusively in neurology departments or in ICU. Using the same operational definition of CSE (seizure lasting > 5 min), and the age cut-off of 16 years old, Kantanen *et al*. found a four-fold increase in incidence of SE compared to the previous ILAE definition (seizure lasting > 30 min) reported earlier in Europe [10].

The mean age around 50 was also reported in Senegal and Thailand [5], [11]. However, other studies have found a lower mean age, particularly in Brazil and Ivory Coast [12], [13]. This could be explained by the difference in methodology. Patient aged ≥ 60 accounted for 37.7% of cases in our study. Studies in Iran and Senegal found 22.4% and 37.6%, respectively, of patients aged ≥ 60 [5], [14]. This high proportion of the elderly could be explained by the epidemiological transition in Africa with a rise of cardiovascular diseases more frequently affecting the elderly. The male predominance of SE was reported by other studies carried out in Africa [1], [7], [15]. The higher frequency of SE in men is probably related to the gender difference of strokes and head trauma related activities (such as motorbike driving). However, some studies have reported a female predominance [14], [16].

In our study, 26.4% of patients had pre-existing epilepsy. Two studies conducted in Nigeria and Madagascar found 23.7% to 28.7% of cases with pre-existing epilepsy [1], [15]. Several studies have reported a higher frequency of patients with epilepsy [12], [17], [18], [19], [20], [21], [22]. In general, people with epilepsy account for 30 to 50% of SE cases [23].

In more than 20% of cases, seizures lasted ≥ 30 min. Cissé *et al*., and Gams *et al*. reported prolonged seizures (more than 30 min) in the majority of cases [2], [5]. The permanent presence of a critical care physician could contribute to lower percentage of prolonged seizures in the MSED of the Douala General Hospital. In addition, there was a marked reduction in the mean time for general physician consultation from 23 min in 2014, to 10 min in this study; and the mean time to first treatment from 26 min in 2014 to 8 min in this study (12 min for first line anticonvulsant) [24]. The risk of occurrence of irreversible neuronal damage increases with the duration of seizure [25]. Focal CSE was observed in more than half of cases. Several studies also found a predominance of focal CSE [12], [16], [17], [19]. However, many authors reported a predominance of generalized seizures [5], [6], [7], [14], [15], [18]. EEG is usually recorded after complete seizure cessation. EEG was performed in less than one third of cases, and the pattern was abnormal in six patients. In Senegal, Gams *et al.* reported that EEG was performed in only 10% of patients and 7% of these patients presented with anomalies [5]. Anyway, the EEG recording should not delay the treatment. Brain imaging had been performed in 92.4% of patients in this study. Data obtained in other developing countries found 66% in Guinea Conakry and 45.4% in Ethiopia [2], [7]. The Douala general hospital has a radiology department with a CT scan and an MRI which operates 24/7. This could also explain the shorter mean time to perform brain imaging (3.5 ± 1.6 hours), compared to Cissé et al., who found a mean time of 24.66 hours [2].

The main etiologies found were: stroke, CNS infections, ASM modifications, brain tumors, brain trauma, and metabolic disorders. For Vignatelli et al., stroke, metabolic disorders, and head trauma were the commonest etiologies [16]. In India, CNS infections, inadequate ASM intake, and stroke were the main etiologies [22]. In Brazil, CNS infections, stroke, metabolic disorders were the reported etiologies [12]. In Sub-Saharan Africa, the commonest causes reported are CNS infections, ASM modifications, metabolic disorders, and stroke [5], [6], [7]. Recent African and Western studies confirm the predominance of cerebrovascular causes [2], [5], [15]. According to Adeloye, the burden of stroke in Africa is high and still increasing [26].

The first-line treatment (Diazepam 10 mg IV repeated once when needed) was administered in about 85% of cases with 75.5% seizure cessation. According to Glauser et al., in adult with CSE, intramuscular (IM) Midazolam, IV Lorazepam, and IV Diazepam are established as efficacious as initial therapy with a superiority of IM Midazolam [8]. In our health facility, Diazepam and Phenobarbital are the injectable ASM frequently available. Cissé *et al*. prescribed as first line treatment Diazepam (66.67%), Clonazepam (26.6%), and Phenobarbital (6.67%) [2]. In China, Chen *et al*. also administered Diazepam and phenobarbital as first line treatment [21]. In Turkey, IV Diazepam combined with IV Phenytoin was the first-line treatment with seizures withdrawal in 75% of cases [27]. For Kumar *et al*., IV Lorazepam was the first line treatment [28]. Lorazepam and Phenytoin are not available in our setting. Due to the frequent interruption of IV Clonazepam, the practice of MSED at the Douala general hospital is to use Diazepam as the first line before switching to Clonazepam if necessary. The second line treatment consisted of IV Clonazepam, and Phenobarbital. IV Phenobarbital or IV Valproate administered as a second line treatment by Poursadeghfard *et al.,* had shown an efficacy of 3.7% and 2.2% respectively [14]. Greater efficacy was reported after administration of IV Phenytoin/Fosfophenytoin [29]. About 9.4% of patients who presented with RSE were admitted, into ICU. Langer and Fontaine reported RSE in 12% of patients with CSE [29]. There is no difference in the incidence of RSE in developing and developed countries. RSE occurs in 29 to 43% of SE cases [30]. In resource-limited settings where access to ASM is limited, injectable diazepam and phenobarbital may be effective option for the management CSE in adults.

The in-hospital mortality was 16.9%. A lower mortality rate was found in Germany by Knake *et al.* (9.4%) and by Coeytaux *et al.* (7.6%) in Switzerland [17], [19]. Vignatelli *et al.* found 39% mortality [16]. In Africa, SE mortality is between 21.3 and 24.8% with a disparity depending on the patient’s age [5], [6], [15]. In developed countries, the use of standardized treatment protocol, the availability of ASM and high standard emergency departments and ICU may contribute to this lower mortality. RSE was statistically associated to death in our study. For Owolabi *et al*., duration of seizure, delay in initiation of treatment and RSE were significantly associated with death [15]. Stupor or coma, NCSE, and age greater than 64 years were considered poor outcome factors, while a history of previous seizures was considered a good outcome factor [30]. Prolonged seizures increase the risk of irreversible neuronal damage and death [25].

## Conclusion

Cameroon is a developing country in Africa where few data are available on the treatment approach to CSE. Our cross-sectional study found the most common cause of CSE was stroke followed by CNS infections. Outcomes were similar to multicenter outcomes using a protocol to guide treatment of CSE, yet despite treatment, one in 10 patients exhibited refractory SE with significant in-hospital mortality. Despite geographic differences and resource limitations, similarities exist between developing and developed countries treating patients with CSE when an organized approach to treatment is followed.

## Declaration of Competing Interest

The authors declare that they have no known competing financial interests or personal relationships that could have appeared to influence the work reported in this paper.
